# Chemical evocation of human cell plasticity—twist of cell fates by small molecules

**DOI:** 10.1093/lifemedi/lnac010

**Published:** 2022-06-28

**Authors:** Ge Liu, Jidong Fu, Nan Cao

**Affiliations:** Department of Genetics and Cell Biology, Zhongshan School of Medicine, Sun Yat-sen University, Guangzhou 510080, China; Departments of Physiology and Cell Biology, The Dorothy M. Davis Heart and Lung Research Institute, Frick Center for Heart Failure and Arrhythmia, The Ohio State University, Columbus, OH 43210, USA; Department of Genetics and Cell Biology, Zhongshan School of Medicine, Sun Yat-sen University, Guangzhou 510080, China

In the past two decades, it has become clear that ‘terminally differentiated’ cells remain certain plastic capacities and can adopt a drastically different fate via activating specific genetic programs. The landmark discovery of induced pluripotent stem cell (iPSC) technology by Takahashi and Yamanaka [1] revealed that this pluripotency of early developmental stage is reversible and can be reestablished in terminally differentiated cells by ectopic expression of master pluripotency transcription factors (TFs). This revolutionary discovery had inspired scientists to explore further whether a combination of specific TFs could switch terminally differentiated cells from one cell type directly into another type without first reversing to pluripotency. It has been identified diverse sets of master regulators, including TFs, microRNAs, RNA-binding proteins, or epigenetic modifiers, that serve as unique ‘cell fate code’ for direct cell fate conversion of different cell types [2]. These direct cellular reprogramming approaches not only evoke a new angle on investigating cell fate determination but also provide alternative resources of desired cell types for disease modeling, drug discovery, and regenerative medicine.

However, the genetic manipulation of master regulator genes is technically very challenging with bioethics safety risks of disrupting genome, which limits its clinical applications. A small molecule-based chemical strategy is one promising alternative approach to generate desired cell types from somatic cells directly without inadvertent genomic modifications of transgenes [3]. In addition to many advantages, such as cell-permeable, reversible, easy to use and standardize, and cost-effective, small molecules can be modified in terms of concentration, duration, structure, and combination; therefore, chemical reprogramming approaches offer tunability and precise temporal control of optimized stage-specific induction conditions to convert cell fate effectively. Noticeably, most successful examples of chemical reprogramming have been made in mouse but become challenging in human somatic cells, because, evolutionarily, human cells have acquired a more complicated and stable epigenetic landscape which accounts for greater refractoriness to reprogramming.

**Figure 1.**
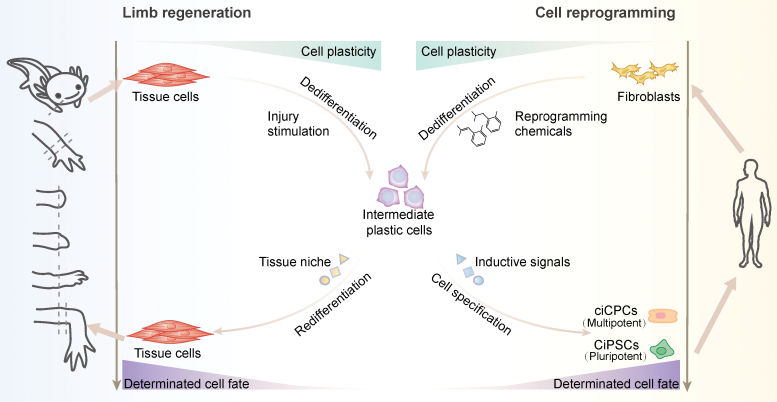
**Chemically reprogramming of somatic cells into expandable CiPSCs and ciCPCs.** Pluripotent or cardiovascular lineage-restricted stem cells can be directly generated by treating human fibroblasts with different combinations of small molecule compounds. Reprogramming compound treatment in human fibroblasts results in the formation of an intermediate plastic state characterized by increased cell proliferation and more open-chromatin conformation at many embryonic developmental genes, enabling their promoters/enhancers to bind effectors of lineage inductive signals. This intermediate plastic state is similar to the dedifferentiated precursors that emerge during axolotl limb regeneration. Thus, this chemical reprogramming strategy in part mimics the endogenous regeneration program to increase cell plasticity and unlock the restricted potential of somatic cells for direct cell fate exchange.

Recently, two publications, Guan et al. [4] and Wang et al. [5], reported the successful reprogramming of human fibroblasts into chemically induced pluripotent stem cells (CiPSCs) and expandable cardiovascular progenitor cells (ciCPCs), respectively. CiPSCs are pluripotent and gain the capacity to differentiate into any cell type in body. While ciCPCs are multipotent and have restricted potential to differentiate into only cardiovascular cells. Both CiPSCs and ciCPCs closely resemble their *in vivo* counterparts in terms of the transcriptional signatures and differentiation capacity. Importantly, both CiPSCs and ciCPCs can be long-term cultured in chemically defined media or even xeno-free culture conditions while faithfully maintaining their undifferentiated phenotype and the pluri- or multipotency potential of differentiation (Fig. 1). These two groundbreaking discoveries offer unlimited autologous and expandable stem cell resources for downstream implications and lay the foundations for developing regenerative therapeutic strategies using chemicals to modulate cell fates in humans.

In most studies of the conventional direct reprogramming of somatic cells, transgenes or chemical molecules induce a direct, one-for-one exchange of cell types, and reprogrammed cells usually cease division right after reprogramming initiation and exit the cell cycle rapidly. It was challenging to obtain progenitor cells that can be extensively expanded. Guan et al. and Wang et al. report the generation of proliferative stem cells that are long-term expandable in chemically defined conditions without oncogene immortalization, making these cells suitable for cryopreservation and abundant enough for subsequent application. Considering their autologous nature, these unlimitedly renewable cells open the possibility for biobanking patient-specific stem cells with careful quality control and may find important uses in personalized cell therapy and precise drug screening and disease modeling. In addition, compared with the previous research of direct lineage conversion in the absence of cell proliferation, reprogramming of human somatic cells into proliferative stem cells is more likely to fully reshape the global epigenome, as complete removal of epigenetic memory of the starting cells is thought to be dependent on cell cycle entry [6].

By using different sets of defined small molecules, both Guan et al. and Wang et al. achieve chemical-based perturbation of the starting cell fate and to recreate a plastic state permissive to certain inductive cues, which in turn enables the further conversion to the target cells. This trajectory somehow mimics the epimorphic regeneration processes in some lower animals (e.g. newts and axolotl), in which terminally differentiated cells at the injury site undergo versatile dedifferentiation and generate a destabilized yet highly plastic intermediated precursors that can re-differentiate to replace lost cells or acquire an alternative cell fate under external inductive signals [7]. It has been asked whether the *in vitro* chemical reprogramming strategy mentioned above can be mimicked and harnessed for *in vivo* regeneration in mammals. More specifically, by treating with reprogramming-inducing pharmaceuticals, cells in damaged tissue may rewrite their fate *in situ* to regenerate organs from within via activation of a regeneration-like program. If achieved, it can potentially overcome the technical hurdles associated with cell transplantation-based therapy, including purification and careful quality control of seeding cells, efficient and safe cell delivery, as well as effective long-term graft survival and integration with the host.

In addition, the chemical reprogramming approaches mentioned above may hold the potential to rejuvenate human cells or reverse aging-related defects without genetic manipulation. A recent study has shown that *in vivo* partial reprogramming by transient induction of reprogramming genes can alter age-associated molecular features and extends the life span of aged mice [8]. Theoretically, transient gene expression can be relatively easily achieved by chemical approaches. As small molecule cocktails developed by Guan et al. and Wang et al. can work similarly as the reprogramming genes to initiate a wave of epigenetic remodeling that activate human somatic cells, energizing their further cell fate conversion, it can be speculated that similar sets of reprogramming chemicals may hold promises to restore a youthful epigenetic signature and delay aging phenotypes when applied *in vivo*. If achieved, it may provide a novel pharmacological strategy that is widely useful in treating various aging-related diseases and disorders.

Despite the tremendous success achieved, many hurdles ahead remain and extensively further optimization is still required for advancing the clinical translation of a chemical reprogramming technique. Firstly, reprogramming efficiency, speed, and quality, as well as rate of success should be further improved to meet the requirement of personalized cell therapy. To achieve this goal, further studies in understanding the mechanism of chemical-based erasure and re-establishment of cell fates will provide valuable information to identify reprogramming barriers and boosters, through which researchers can further optimize the small molecule formula. Encouragingly, many cutting-edge techniques, such as single-cell multi-omics, CRISPR-based genome-wide genetic screening, and high-content chemical screening, have been proved to be tremendously successful in transgene-based cell reprogramming studies [9]. They have identified alternative reprogramming routes as well as the pathways and mechanisms that control such progress, which must be inhibited or activated to obtain the desired cell type and reduce heterogeneity. It is conceivable that these research strategies and methods can be similarly applied to a chemical reprogramming context and continue to play a critical role in dissecting the underlying molecular mechanisms. Secondly, it needs to develop early diagnosis methods to predict reprogramming success because chemical reprogramming of human cells usually has slower kinetics and takes months for complete cell conversion and expansion, and donor cells from people of various ages show significant differences in reprogramming efficiency, yields, and degrees of genome instability. If achieved, unsuccessful reprogramming experiments can be terminated as early as possible to save time and effort. This will further require deepened knowledge of the regulatory mechanisms during early chemical reprogramming, through which key morphological and molecular features of reprogramming-prone or reprogramming-resistant cells can be identified and harnessed as trackable markers. Once again, single cell-based imaging and molecular analysis methods, as well as machine learning-aided image recognition technologies, may greatly facilitate the realization of the above goals. Thirdly, despite the tremendous potential of chemical reprogramming for *in situ* organ repair, much effort is needed to identify a suitable and efficient administration approach to targetedly deliver the small molecules for *in vivo* reprogramming. Suitable controlled drug delivery systems [10], such as microsome entrapment, nanocarriers, and nanocrystals, may help to achieve this goal and warrant further investigations.

In summary, it is believed that cross-disciplinary collaborations and applications of many advanced techniques in biology and bioengineering will enable us to overcome the difficulties associated with the translation of chemical reprogramming approaches and open new avenues for regenerative medicine.
